# Red Blood Cell Distribution Width, Neutrophil-to-Lymphocyte Ratio, and In-Hospital Mortality in Dyspneic Patients Admitted to the Emergency Department

**DOI:** 10.1155/2020/8839506

**Published:** 2020-06-18

**Authors:** Li Yan, Zhi-De Hu

**Affiliations:** ^1^Department of Respiratory and Critical Care Medicine, The Affiliated Hospital of Inner Mongolia Medical University, Hohhot, China; ^2^Department of Laboratory Medicine, The Affiliated Hospital of Inner Mongolia Medical University, Hohhot, China

## Abstract

Red blood cell distribution width (RDW) and neutrophil-to-lymphocyte ratio (NLR) have shown a prognostic value in various clinical settings. We aimed to investigate the association between RDW, NLR, and in-hospital mortality in patients with dyspnea. In this retrospective study with the Medical Information Mart for Intensive Care III database (version 1.4), adult patients who came to the emergency department with dyspnea were included. Patients' comorbidities, hematological parameters within the first 48 hours after admission to the emergency department, and in-hospital mortality were extracted. The relationships between RDW, NLR, and in-hospital mortality were analyzed with the receiver operating characteristic (ROC) curve analysis and multivariate logistic regression model. We found that hospital survivors had significantly lower NLR than those who died. However, RDW was not significantly increased in patients who died during the hospitalization. The area under the ROC curve of NLR for predicting in-hospital mortality was 0.62. On multivariate analysis, NLR was not independently associated with in-hospital mortality. On further analysis, lymphocyte percentage was independently associated with in-hospital mortality, with an odds ratio of 0.56. Therefore, we concluded that RDW and NLR are not reliable parameters to predict in-hospital mortality in critically ill patients admitted to the emergency department with dyspnea.

## 1. Introduction

Dyspnea is one of the most common symptoms at presentation to the emergency department (ED) [1]. Dyspnea can be the initial clinical manifestation of many disorders, such as congestive heart failure (CHF), pneumonia, acute myocardial infarction (AMI), and sepsis [2]. Patients with dyspnea are found to have higher mortality and readmission rate [3], and early risk stratification is crucial to improve patient outcomes [1].

Red blood cell distribution width (RDW) is a routinely reported hematology parameters that can be automatically measured by modern hematological analyzers [4]. It is a measure of heterogeneity in the size of circulating red blood cells (RBC). In the past, RDW has been used for differentiating thalassemia and iron deficiency anemia [5]. However, recent studies have revealed that RDW is also a useful test for estimating the prognosis or disease activity of various cancers [6, 7], thyroiditis [8], gastrointestinal disorders [9], cardiovascular diseases [10–13], critical illness [14–16], and autoimmune diseases [17, 18]. This may be due to the fact that RDW is an inflammatory parameter [17], and inflammatory response is involved in the progression of various disorders. To present, two studies have investigated the prognostic value of RDW in unselected dyspneic patients [19, 20], but with varying results. Therefore, further evidence is needed to clarify the prognostic value of RDW in dyspneic patients.

Similarly, the neutrophil-to-lymphocyte ratio (NLR) is an inflammatory parameter that can be easily calculated from routinely available hematology parameters [21]. Similar to RDW, NLR has been found to be a prognostic factor or diagnostic marker in various diseases, such as pregnancy-related disorders [22], thyroiditis [23], cancers [24, 25], and cardiovascular diseases [26–29]. However, the role of NLR in patients with dyspnea remains unknown. Because NLR is an inflammatory parameter and inflammatory markers are associated with prognosis in the dyspneic patients [30], we hypothesized that NLR could be used for risk stratification in dyspneic patients. In this study, we aimed to investigate the prognostic value of RDW and NLR in dyspneic patients.

## 2. Materials and Methods

### 2.1. Database and Patients

This is a retrospective study with MIMIC-III (Medical Information Mart for Intensive Care III) database (version 1.4). MIMIC III is a large, freely accessible critical care database comprising 38,645 adults and 7,875 neonates who were admitted to the intensive care unit (ICU) of Beth Israel Deaconess Medical Center between 2001 and 2012 [31, 32]. The Institutional Review Boards (IRB) of the Massachusetts Institute of Technology (MIT) approved the establishment of MIMIC III. One of the authors (ZD Hu, certification number: 1678079) has passed a web-based course and was approved for extracting data from MIMIC III for research purposes. Informed consent was waived because all data are from a publicly available database.

The inclusion criteria of this study were as follows: (i) patients who visit the emergency department (ED) complains of dyspnea; (ii) RDW determined within 48 hours after emergency department admission. Patients with aged <18 years were excluded.

### 2.2. Data Extraction

The clinical and demographical data of the patients were stored in 26 tables and can be extracted with structured query language (SQL). We extracted the following data from MIMIC III: age, gender, ethnicity, hematology parameters, comorbidities [33], and final diagnosis. Only the hematological parameters determined within 48 hours at the time of presentation after ED admission were included. For patients with more than one hematology parameters during the first 48 hours after ED admission, only the first result was studied. The SQL script used to extract comorbidities was available from the GitHub website (https://github.com/MIT-LCP/mimic-code/tree/master/concepts, date of access: January 2019) [33]. We used the keywords “dyspnea” and “shortness of breath” to search dyspneic patients in the ADMISSIONS table of MIMIC III.

### 2.3. Statistical Analysis

Continuous data were presented as mean and standard deviation (SD). The normal distribution of continuous variables was tested using the Kolmogorov-Smirnov test. Mann-Whitney *U* test or Student's *t* test was used for continuous data comparison between two groups, if appropriate. A Chi-square test was used for categorical variable comparison. Receiver operating characteristic (ROC) curve analysis was used to assess the predictive accuracy of hematology parameters for in-hospital mortality. The multivariable logistic regression model was used to analyze the association between hematology parameters and in-hospital mortality. All statistical analyses were performed using R (version 3.5.0 (The R Foundation for Statistical Computing)), and *p* value < 0.05 was considered as statistically significant.

## 3. Results

### 3.1. Characteristics of the Patients

**Figure 1:**
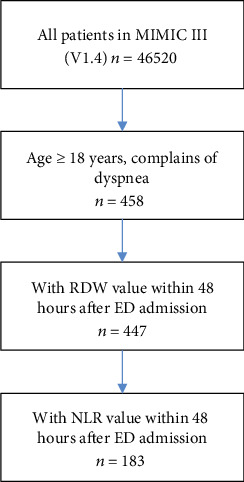
Flow chart for subject identification and inclusion.

Figure 1 is a flowchart depicting the patient selection process. Finally, 447 patients with a RDW value were included in the present work, and 183 of them have a NLR value. The clinical characteristics of the patients are summarized in Table 1. Notably, more than half of the patients had comorbidity of CHF, and one-fourth of patients had renal failure. Compared with hospital survivors, patients who died in hospital were older and had significantly higher white blood cell (WBC) count and NLR, as well as lower lymphocyte percentage. However, we failed to find significantly higher RDW in the patients who died in the hospital as compared to survivors (*p* = 0.690).

### 3.2. Accuracy of RDW and NLR for In-Hospital Mortality

**Figure 2:**
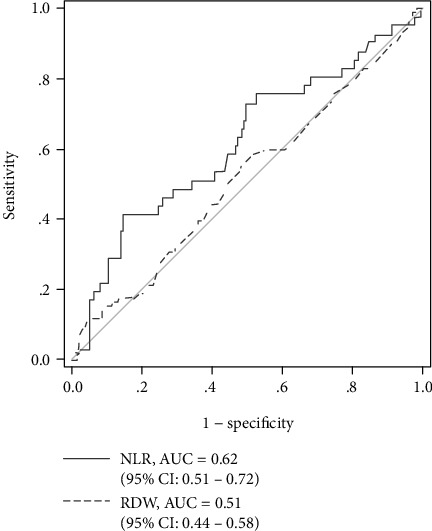
Receiver operating characteristic (ROC) curve of RDW and NLR for predicting in-hospital mortality.

Figure 2 shows ROC curve of RDW and NLR for in-hospital mortality. The area under curve (AUC) of NLR was 0.62 (95% CI: 0.51–0.72, *p* = 0.024). The AUC of RDW was not statistically significant (AUC = 0.51; 95% CI: 0.44–0.58; *p* = 0.690). In addition, we found that the AUC of the lymphocyte percentage was 0.64 (95% CI: 0.54–0.74; *p* = 0.008).

### 3.3. Multivariate Analysis

Next, we performed a multivariable logistic regression analysis to study the association between NLR, lymphocyte percentage, and in-hospital mortality. In a multivariate model containing age, CHF, hypertension, and log-transformed NLR, we did not find an independent association between NLR and in-hospital mortality (OR = 1.42; 95% CI: 0.98–2.10; *p* = 0.07). Interestingly, in a model containing log-transformed lymphocyte, age, hypertension, and CHF, log-transformed lymphocyte percentage was independently associated with in-hospital mortality (OR = 0.56, 95% CI: 0.35–0.88, *p* = 0.01). We did not perform a multivariate model with age, CHF, and both log-transformed NLR and lymphocyte percentage because of the collinearity between NLR and lymphocyte percentage.

## 4. Discussion

There are some interesting findings in this study. First, RDW was not associated with in-hospital mortality in dyspneic patients. Second, the predictive accuracy of NLR for in-hospital mortality is fair, it was not independently associated with in-hospital mortality after age, and the comorbidity of heart failure was adjusted. Third, increased lymphocyte percentage was independently associated with low in-hospital mortality.

Previous two studies have indicated that RDW was independently associated with 30-day and 1-year mortality in dyspneic patients [19, 20]. However, the prognostic value of RDW was not found in this study. The inconsistency between the present and previous studies may have two possible explanations. First, all of the patients in the present study have critical illness and are admitted to the intensive care unit after ED admission. Notably, the in-hospital mortality in the present study was 19.2% (86/447), while the 30-day and 1-year mortality in previous studies was 9.5% [19] and 12.5% [20], respectively, indicating that the patients in the present study had severe disease. Inflammatory response has been proposed to be a mediator between RDW, NLR, and prognosis of dyspneic patients. In dyspneic patients with critical illness, strong inflammatory response is common, and therefore, the effects of inflammatory markers on the prognosis of patients may be attenuated. Second, the mean hemoglobin level in the present study was 107 g/L, which seems lower than that in previous studies [19, 20]. It is widely accepted that RDW is greatly affected by hemoglobin levels [34]. Indeed, the mean RDW level present was 15.9%, which seems higher than that in previous studies. Taken together, these results indicate that RDW may have a limited prognostic value in dyspneic patients with low hemoglobin and critical illness.

To the best of our knowledge, this is the first study investigating the prognostic value of NLR in dyspneic patients. We found that the predictive accuracy of NLR and lymphocyte percentage for in-hospital mortality is mild, with AUCs of 0.62 and 0.64, indicating that NLR and lymphocyte percentage may not be useful prognostic factors in dyspneic patients. Indeed, NLR was not independently associated with in-hospital mortality in a multivariable logistic regression model. However, considering that the patients in the present study are severely diseased, further studies with common dyspneic patients are needed to evaluate the prognostic value of NLR and lymphocyte percentage.

The present study has some limitations. First, this is a retrospective study with a small sample size. Second, to define dyspneic patients, we used the keywords “dyspnea” and “shortness of breath” to search the variable of DIAGNOSIS in the ADMISSIONS table. The variable DIAGNOSIS provides a free text and preliminary diagnosis for the patient on hospital admission. It is usually assigned by the admitting caregivers and does not use a systematic ontology. Therefore, some dyspneic patients may have been missed by the search keywords. In addition, because some clinical data are missing in the MIMIC III database, the confounding factors adjusted in multivariable regression analysis are limited.

In conclusion, we found that both NLR and RDW have a limited value for predicting in-hospital mortality in severely diseased patients with dyspnea. Giving the retrospective design and small sample size of the present study, further studies with a large sample size, full adjustment, and prospective design are needed to validate the findings of the present study.

## Figures and Tables

**Table 1:** Characteristics of the subjects.

	All		Survivor		Nonsurvivor		*p*
	*n*	Results	*n*	Results	*n*	Results	
Age (years)	447	68 (56–79)	361	66 (56–78)	86	75 (61–82)	0.003
Gender (M/F)	447	213/234	361	172/189	86	41/45	1.000
Ethnicity (white/others)	447	320/127	361	246/115	86	74/12	0.001
Hematology							
WBC (10^9^/L)	447	8.7 (6.4–12.9)	361	8.5 (6.4–12.0)	86	10.6 (6.3–15.9)	0.034
Neutrophil (%)	184	84 (74–90)	143	84 (74–90)	41	85 (76–91)	0.398
Lymphocyte (%)	183	9 (5–16)	142	10 (6–16)	41	6 (4–10)	0.008
NLR	183	9.1 (4.7–17.2)	142	8.3 (4.5–15.1)	86	11.4 (7.9–25.2)	0.024
Hemoglobin (g/L)	447	105 (92–120)	361	106 (92–120)	86	101 (89–118)	0.362
RDW (%)	447	15.5 (14.2–17.2)	361	15.4 (14.2–17.2)	86	15.7 (14.2–17.2)	0.690
MCV (fl)	447	90 (86–94)	361	90 (86–95)	86	90 (85–94)	0.358
MCH (pg)	447	29.6 (27.9–31.4)	361	29.6 (28.0–31.5)	86	29.6 (27.8–30.8)	0.230
MCHC (g/L)	447	327 (317–338)	361	328 (318–339)	86	325 (317–336)	0.336
Comorbidity							
CHF (Y/N)	447	230/217	361	199/162	86	31/55	0.002
Liver disease (Y/N)	447	45/402	361	32/329	86	13/73	0.125
Renal failure (Y/N)	447	117/330	361	99/262	86	18/68	0.218
Hypertension (Y/N)	447	247/200	361	209/152	86	38/48	0.029
Cardiac arrhythmias (Y/N)	447	174/273	361	138/223	86	36/50	0.619
Valvular disease (Y/N)	447	74/373	361	62/299	86	12/74	0.575
CPD (Y/N)	447	193/254	361	158/203	86	35/51	0.693
Hypothyroidism (Y/N)	447	63/384	361	55/306	86	8/78	0.212
Final diagnosis							
Heart failure	447	65	361	56	86	9	0.233
Pneumonia	447	35	361	28	86	7	0.905

WBC: white blood cell; NLR: neutrophil-to-lymphocyte ratio; RDW: red blood cell distribution width; MCV: mean corpuscular volume; MCH: mean corpuscular hemoglobin; MCHC: mean corpuscular hemoglobin concentration; CHF: congestive heart failure; CPD: chronic pulmonary disease.

## Data Availability

The data used to support the findings of this study are available from the corresponding author upon request.
